# A Case of Giant Uterine Lipoleiomyoma Simulating Malignancy

**DOI:** 10.1155/2015/926961

**Published:** 2015-07-22

**Authors:** Erbil Karaman, Numan Çim, Gülay Bulut, Gülhan Elçi, Esra Andıç, Mustafa Tekin, Ali Kolusarı

**Affiliations:** ^1^Department of Obstetrics and Gynecology, Yuzuncu Yil University, 65000 Van, Turkey; ^2^Department of Pathology, Yuzuncu Yil University, 65000 Van, Turkey

## Abstract

*Introduction*. Uterine leiomyoma is the most common benign pathology in women and lipoleiomyoma is an extremely rare and specific type of leiomyoma. Here, we report an unusual case of giant pedunculated subserous lipoleiomyoma misdiagnosed preoperatively as leiomyosarcoma. *Case*. A 45-year-old woman admitted to our gynecology outpatient clinic for complaints of abdominal distention, tiredness, and pelvic pain for the last 6 months. Sonography and abdominal magnetic resonance imaging (MRI) showed a giant semisolid mass that filled whole abdominal cavity from pelvis to subdiaphragmatic area. A primary diagnosis of uterine sarcoma or ovarian malignancy was made. On operation, total abdominal hysterectomy with a pedunculated mass of size 30 × 23 × 12 cm and weighing 5.4 kg and bilateral salpingo-oophorectomy were performed. The histopathology revealed a lipoleiomyoma with extensive cystic and fatty degeneration without any malignancy. *Discussion*. The diagnosis of leiomyoma is done usually with pelvic ultrasound but sometimes it is difficult to reach a correct diagnosis especially in cases of giant and pedunculated lipoleiomyoma that included fatty tissue which may mimick malignancy. *Conclusion*. Subserous pedunculated giant lipoleiomyoma should be kept in mind in the differential diagnosis of leiomyosarcoma or ovarian malignancy.

## 1. Introduction

Uterine leiomyomas are the most commonly seen gynecologic tumors and their prevalence is stated as 25–40% in reproductive age [1]. The fibroids originate from the smooth muscle cells of uterine wall. Its size varies from microscopic to giant and they can be submucosal, intramural, or subserous location. Huge uterine myomas are exceedingly rare [2]. Lipoleiomyoma is a benign variant of leiomyoma and is composed of mature smooth muscle cells and adipocytes with an incidence ranging from 0.03% to 0.2%. The exact etiology of fibroids is still unknown but it is linked with the role of estradiol and growth factors [3]. However, fatty metamorphogenesis of the smooth muscle cells of leiomyomas is the most likely cause for the development of lipoleiomyoma [4]. The complaints of fibroids can be menstrual disturbances, pelvic pain, constipation, micturition problem, or some effects on fertility such as miscarriage and preterm labour. The diagnosis of fibroids is made with ultrasound or MRI with a good accuracy. However, in case of pedunculated giant myoma with thin stalk and fatty cystic degeneration, the diagnosis is difficult and can be misdiagnosed as uterine sarcoma or ovarian malignancy. The treatment options vary from expectant management of small and asymptomatic fibroids to surgical therapy especially in case of giant ones.

## 2. Case

**Figure 1:**
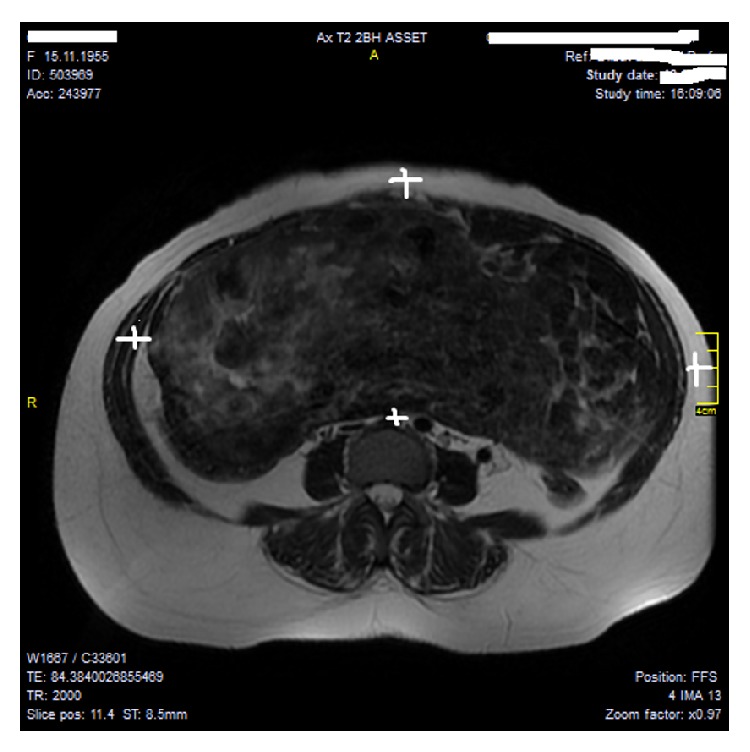
The figure shows MRI image of giant pelvic mass filling whole abdominal cavity with heterogenous and semisolid appearance (corresponding the image between four white callipers).

**Figure 2:**
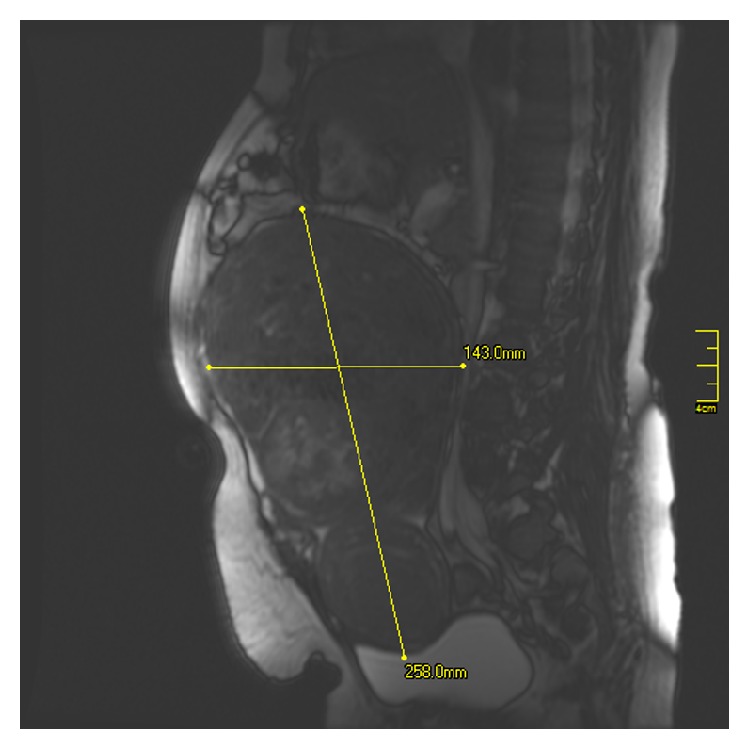
The figure shows the sagittal view of mass that is filling abdominopelvic cavity.

A 45-year-old premenopausal multiparous woman was admitted to our hospital's outpatient gynecology clinic with complaints of lower abdominal pain and abdominal distension for the last 6 months. On detailed anamnesis, the patient had noticed a mass in her abdomen for 3 months and a gradually increasing pain with easy tiredness. She had four previous vaginal deliveries with no abdominal surgical operation. Her medical history was remarkable for 10 years of type II diabetes and hypercholesterolemia and had no history for family member with genital malignancy. She had no complaints related with menstrual bleeding. On physical examination, her vital signs were normal and abdominal palpation revealed a distended abdomen with palpable hard, solid mass filling whole abdominal cavity which cannot be lateralized. No abdominal rebound or tenderness was observed. The speculum examination showed a normal uterine cervix and vagina but fornices were full on pelvic examination. Initially, a transvaginal ultrasound was applied and showed a large, solid, and complex mass in pelvic cavity which extended to subdiaphragmatic area and its origin could not be found. An MRI scan of abdomen showed that a large solid mass with somewhere in cystic and fatty content, approximately 33 × 17 × 25 cm in size, which could not be separated from uterus was noticed (Figures 1 and 2). No normal ovaries were detected.

**Figure 3:**
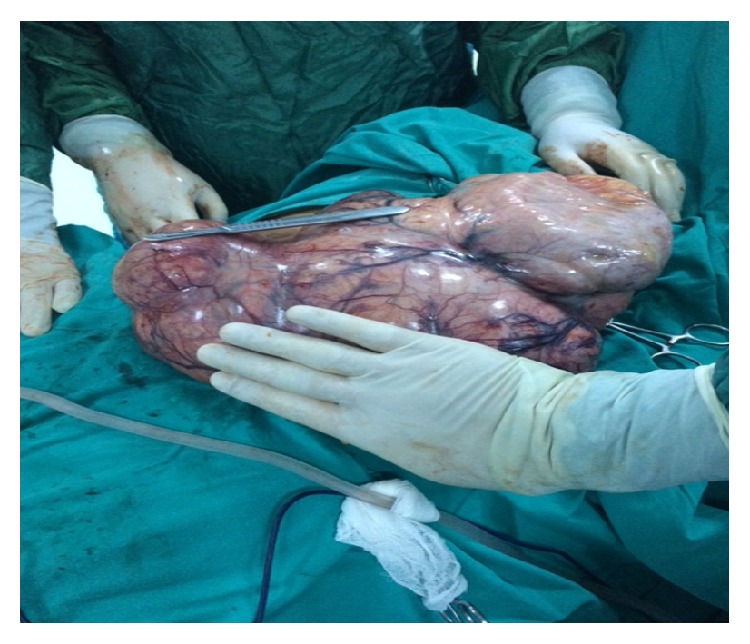
The figure shows intra-abdominal view of multilobulated white-yellowish coloured mass with size of 30 × 23 × 12 cm and 5.4 kg.

Laboratory examinations for whole blood count, liver function tests, coagulation parameters, urea, creatinine, and serum electrolytes were in normal limits. Blood glucose, HgA1c level, and cholesterol levels were higher than normal limits. The serum level of cancer antigen-125 was detected to be high as 210 mIU/ml. So in the light of these clinical findings from ultrasound, MRI examinations and laboratory findings, we thought that leiomyosarcoma is the most likely diagnosis. We planned surgery for the patient and a midline xiphopubic vertical incision was made. At laparotomy, on inspection, a giant multilobulated solid mass with white-yellowish colour was noted and we thought a huge mass of ovarian malignancy, however when we put up the mass from abdomen out, then we saw that it was a pedunculated giant subserosal myoma with a thin stalk (Figure 3). Firstly, we excised the mass from uterus and sent it to frozen section. Total abdominal hysterectomy and bilateral salpingo-oophorectomy were carried out. The frozen section revealed myoma uteri without malignancy. A drain was put into the pelvis and the surgery was completed. The drain was removed in second postoperative day and the patient was discharged 7 days after the operation with no complication.

**Figure 4:**
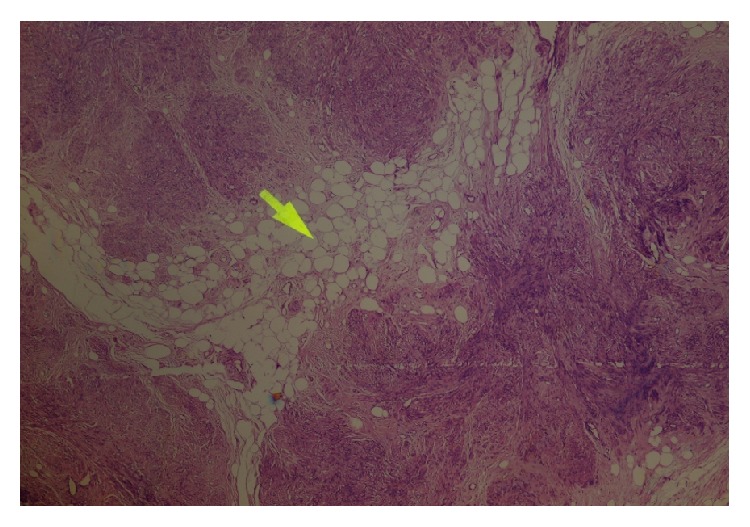
The figure shows spindle-shaped smooth muscle cell proliferation admixed with mature adipocytes (arrow) (HE&40).

Macroscopically, pathologic examination revealed a multilobulated solid mass measuring 32 × 23 × 12 cm showing the appearance of leiomyoma. The microscopic examination showed lipoleiomyoma of uterus with extensive cystic and fatty content as admixed with mature adipocytes without histologic signs of malignancy (Figure 4). Both ovaries and endometrial cytology were detected to be benign as normal ovary and proliferative endometrium.

## 3. Discussion

Leiomyomas are benign tumors of uterine wall and accounting in approximately one-third of women of reproductive age [5]. These benign tumors have a spectrum of clinical manifestations including pelvic pain, increased or abnormal menstrual bleeding periods, infertility problem, or pressure effects on surrounding organs like bladder or rectum. Although the exact etiology is unclear, hormonal stimulation by estrogen, and possibly progesterone, has been suggested as a possible cause [5]. The clinical symptoms vary according to its size which may be microscopic or giant and its location. Based on their location, they are classified as submucosal, intramural, or subserosal. The subserosal myomas may have a thin stalk which cannot be differentiated from uterus and simulate ovarian malignancy [6]. In case of pedunculated giant myomas, the need of blood supply increases over time as they enlarge resulting in various types of degenerations such as hyaline, cystic, red, or calcific-dystrophic degeneration. Hyaline degeneration is the most common type of degeneration, seen in up to 60% of cases. Uterine lipoleiomyoma is a rare and specific type of leiomyoma with a considerable amount of adipocytes [7]. It is reported that lipoleiomyoma arises from metaplasia (neometaplasia) of immature perivascular pluripotent mesenchymal cells or derived from direct metaplasia of the smooth muscle cells of leiomyoma to adipocytes [8].

There are numerous diagnostic modalities for leiomyoma, leiomyosarcoma, or ovarian malignancy including ultrasonography, MRI, computed tomography, hysteroscopy, and saline infusion sonography. But none of these diagnostic imaging modalities can differentiate the benign and malignant growths exactly without confirmation by the pathological examination. Like this, as a blood test, the cancer antigen-125 is a useful marker in diagnosis of malignant ovarian mass but it can be seen in high levels also in uterine fibroids as well as in other benign gynecologic diseases. Ultrasonography is the preferred imaging tool for diagnosis of the initial evaluation after bimanual examination because of its wide availability, being inexpensive, and least invasive character. On ultrasonography, only those parts of the tumor that are close to the probe can be demonstrated clearly. Although uterine mass containing fat can be diagnostic of lipoleiomyoma in ultrasonography, MRI is an important imaging tool for precision for identification, number, and location of these tumors and can exclusively show the fat content within the tumor as well as differentiation from an adnexal mass especially in cases of giant mass which could not be differentiated from an adnexal malignancy [4]. In our case, based on MRI findings, we could not differentiate the pedunculated myoma which had a thin stalk and fatty content from leiomyosarcoma or adnexal malignancy. Therefore, in case of a giant mass that filled whole abdominal cavity, even MRI cannot differentiate its origin exactly.

Pelvic mass during perimenopausal state should be clarified and majority of these are benign gynecologic conditions including primarily uterine fibroids. In case of a giant pelvic mass, the diagnosis is difficult and frequently leads the physicians to suspect favouring the malignant growths of ovarian tissue or leiomyosarcoma. A color Doppler flow can be used to differentiate the malignant and benign ovarian tumors but it cannot be applied to differentiate the lipoleiomyoma and uterine leiomyoma.

As far as we know from literature, there are some reports about giant myomas including lipoleiomyoma which have different clinical scenarios such as in a case report by Akbulut et al. reporting a case of symptomatic giant lipoleiomyoma of the uterine corpus that may be associated with diabetes mellitus and hypothyroidism [9]. In a recent case study by Aydin et al. a 58-year-old postmenopausal woman who had a large cystic myoma measuring 33 × 20 × 18 cm mimicking an ovarian malignancy was reported. They concluded that pedunculated leiomyomas should be considered in the differential diagnosis of a multilocular and predominantly cystic adnexal mass [10]. So leiomyomas can be easily diagnosed on imaging in cases of typical appearances but degenerative changes or fatty content like in lipoleiomyoma may lead to change in its images and can cause difficulty and confusion in diagnosis. Leiomyomas have been misdiagnosed as adenomyosis, hematometra, uterine sarcoma, and ovarian masses and also differential diagnosis of lipoleiomyoma includes angiolipoma, angiomyolipoma, atypical lipoma, and liposarcoma. In our case, we thought the huge mass initially to be leiomyosarcoma and secondly to be ovarian malignancy even just after the intra-abdominal inspection of mass before delivering it from abdominal cavity to the outside during operation.

It was reported that uterine lipoleiomyoma may be associated with metabolic diseases including hyperlipidemia, hypothyroidism, and diabetes mellitus [8]. Lin and Hanai reported that changes in lipid metabolism and other nonlipid mechanisms occurring during menopause might play an important role in the development of lipomatous changes in leiomyoma. In our case, the patient had diabetes and hypercholesterolemia which can be a potential source for increase of plasma lipids and the fatty infiltration of smooth muscle cells.

There are numerous established managements of uterine fibroids and also of lipoleiomyoma including one or a combination of the following as expectant therapy, surgery, medical or hormonal treatment, myolysis, and uterine artery embolization. The treatment should be individualized according to many factors including the patient age, fertility status, the severity and type of symptoms, suspicion of malignancy, the location and size of myomas, and desire of patient. The surgery is most frequently preferred for treatment of giant leiomyomas. The surgical approach of these giant tumors concerns some intraoperative technical difficulty such as the increase of blood loss, any injury to adjacent organs due to dense intestinal adhesions, or anatomical change of ureters because of huge mass within the pelvic cavity.

In conclusion, lipoleiomyoma is a rare and specific type of uterine leiomyoma and it can be diagnosed easily with examination and diagnostic modalities. However, in case of giant myomas the diagnosis can be difficult and masquerading as a malignant adnexal mass especially if there is fatty degeneration and pedunculated myoma. So the physicians must be aware of those giant pedunculated lipoleiomyomas in the differential diagnosis of semisolid and multilocular pelvic masses.
